# Christopher Robert Pemberton

**Published:** 1822-09

**Authors:** 


### OBITUARY.

On the 24th of July, at Fredville, in Kent, (the seat of his brother-in-law,) CHRISTOPHER ROBERT PEMBERTON, M.D. F.R.S. &C.

Christopher Robert, fifth son of the Rev. Jeremy Pemberton, and great-grandson of Francis Lord Chief-Justice Pemberton, was born at Trumpington, in Cambridgeshire, on the 8th of November, 1765. He was educated under the Rev. Dr. Lawrence, at the school of Bury St. Edmund's, and afterwards removed to Caius College, Cambridge; where, after having passed through the usual course of studies at that university, he took the degree of Doctor of Physic, July 1794; he was soon after admitted Fellow of the Royal College of Physicians; and elected one of the physicians to St. George's Hospital, in 1800. The professional merits of Dr. Pemberton were of the first order, and he was remarkable for the urbanity of his manners. About the year 1801, he began to be extensively employed in practice, which the publication of his excellent work on Diseases of the Abdominal Viscera contributed greatly to increase.—When at the height of his reputation, and in the full career of a practice seldom equalled, and perhaps never surpassed, he experienced an attack of that formidable disease the tic douloureux. His subsequent sufferings from repeated paroxysms of this disorder, which gradually undermined his health, and rendered him at length incapable of continuing the exercise of his profession, are too well known, and too recent, to render it necessary for us to dwell upon them here. We join in the general regret at the loss of an excellent and honourable physician and amiable man, and offer our tribute of compassion for the almost unexampled sufferings of an individual, supported for several years with exemplary patience and fortitude, which have deprived the profession of physic of a distinguished ornament.*

On Saturday, the 3d of August, at Oxford, in the 58th year of his age, Sir CHRISTOPHER PEGGE, knight, Regius Professor of Physic at that university.

---

* We hope to be able, in a future Number, to give some particulars of Dr. Pemberton's interesting case.

